# Water pipe smoking: an emerging trend with detrimental consequences

**DOI:** 10.11604/pamj.2014.17.200.2991

**Published:** 2014-03-13

**Authors:** Henry Nyongesa, Jacob Adegu

**Affiliations:** 1 University of Nairobi, School of Medicine, Nairobi, Kenya

**Keywords:** Water pipe, smoking, tobacco

## Commentary

Over the past decade, water pipe smoking has spread globally at an alarming rate. In the Middle East particularly, prevalence estimates of smoking hookah have already surpassed those of cigarette [1]. What captures the magnitude of the trend on a world wide scale is the Global Youth Tobacco survey commissioned in 1999 to provide up to date data on tobacco use among ages 13-15 in 95 countries [2].

Though diverse geographic settings hold different designs of water pipe, the defining feature in the set up is the passage of smoke through water. This has been erroneously referred to as filtering. When flavored tobacco is burnt in charcoal or coal, the smoke passes through the body to the bowl containing water; and then to the smoker through a pipe. What tends to encourage smokers is the false perception emanating from the “filtering” process that is assumed to detoxify smoke contents.

But what factors may be contributing to this wide spread use of water pipe? This question has elicited massive interest as evidenced by an array of surveys performed principally in the Middle East [3–5].

Generally, these factors fall into various domains of human life. From a social perspective, it has been noted through a comparative study that while cigarette smoking is frowned upon, there is more acceptance especially for women to smoke the water pipe [6]. Additionally, this habit is perceived as a means of communal association. It is not uncommon for visitors to be offered a water pipe as an ice breaker. This is replicated even in hospitality settings where clients enquire first on the availability of a water pipe before other services can be rendered [7].

Importantly, the competitive edge of water pipe over cigarette in addition to perceived safety is introduction of factory sweetened flavours. A great majority of water pipes are filled with a variety of flavours for which the partakers obtain a sense of relaxation from demanding lifestyles [8]. It also makes economic sense as water pipe smoking is usually a communal event with expenses customarily shared among smokers [9].

Despite popularity of water pipe, most smokers are ignorant of deleterious health consequences associated with the habit. A matched analysis of toxic components of water pipe and cigarette dwarfed the perceived safety of the former. At the outset is the dramatic exposure to more smoke and carbon monoxide levels of up to four times that found in cigarettes [10]. The polycyclic aromatic hydrocarbons, napthylamines, nitrosamines, and primary aromatic amines have also been isolated in greater quantities [11].

While it has been firmly established that cigarette smoking is a major risk factor for numerous cancers, there has not been long term substantial studies to assess the role of water pipe in cancer causation. However, emerging data from available studies point to a clearly worrying state that warrants urgent attention of public health activists [12–15].

In light of a severe toxicity profile, it can be argued that there will be an unprecedented increase in lung cancer cases. Interestingly, a systematic review by Elie Aki and colleagues could not establish if some of the malignancies associated with cigarette smoking were also associated with water pipe. These included oral dysplasia and cancers of urinary bladder, esophagus and nasopharynx [16]. Nevertheless converging evidence from gene oncology studies remarkably reveals the central role of water pipe smoking in increasing mitotic index, micronuclei, chromosomal aberrations as well as satellite associations [17, 18]. Indeed, these findings indicate that smoking of water pipe just like cigarette, is a major risk for cancer.

Arguably, cigarette and the latest entrant; water pipe smoking are twin disasters that will continue plaguing public health policymakers from the continent. These habits are at the inner core of exacerbating the burden of non communicable disease in less endowed countries of Africa and Asia, which already are reeling from effects of infectious diseases. Drawing from benefit of hindsight that African youth culture is easily shaped by trends in the East and West, it is highly likely that water pipe smoking will be commonplace.

Granted, almost all governments have laws prohibiting public use of tobacco products; it is the lackluster manner of implementation that has slowed war against this publicly repugnant behavior. Even more disturbing is failure to recognize that water pipe is another means of smoking tobacco. It should be appreciated that although the laws are flagrantly flouted, even passive smoking poses grave consequences to the public. Notwithstanding the limitations, we believe that there are abundant opportunities for the continent to engage in concerted efforts to curb this scourge before it spirals out of control.

The simplicity in dealing with this public health hazard can be initiated through a multipronged approach. Consistent public health education on dangers of smoking is a stepping stone to halting the trend as attested in a variety of landmark studies [19]. As paucity of knowledge is one of the contributing factors, education tailored and delivered in an emphatic way goes a great way in reducing the prevalence of tobacco smoking in whatever form. Health care providers should be at forefront in liberating the public from mythological “feel good effects” posed by smoking.

Secondly, it is incumbent upon governments to decisively deal with the menace through implementation of policies to the latter. They should resist underhand deals, so well perfected by large tobacco manufacturers, to prevent the disastrous descent into health anarchism. For the expenses involved in management of patients with cancer and other non-communicable diseases attributable to smoking are agonizing for any government or individual to bear on long term scale. Therefore, the buck does not even stop at formulating tangible preventive measures but rather in rapidly and sustainably implementing them.

In conclusion, water pipe smoking has lately been embraced especially in developing countries oblivious of its more grave effects compared to cigarette. Primarily, the fast growing popularity is hinged on varied factors. We posit that if the habit is not addressed early through effective policy formulation and implementation, then public health systems under African ambit should brace themselves for dire consequences in future.
